# Supplementary material for: Deciphering the Effects of the PYCR Family on Cell Function, Prognostic Value, Immune Infiltration in ccRCC and Pan-Cancer
Source: Int J Mol Sci. 2024 Jul 25;25(15):8096. doi: 10.3390/ijms25158096 (PMC11311831; doi:10.3390/ijms25158096)
Supplement: Supplementary file 1 [file ijms-25-08096-s001.zip › ijms-3120948-supplementary.pdf]

Supplementary Material

1. Supplementary Figures

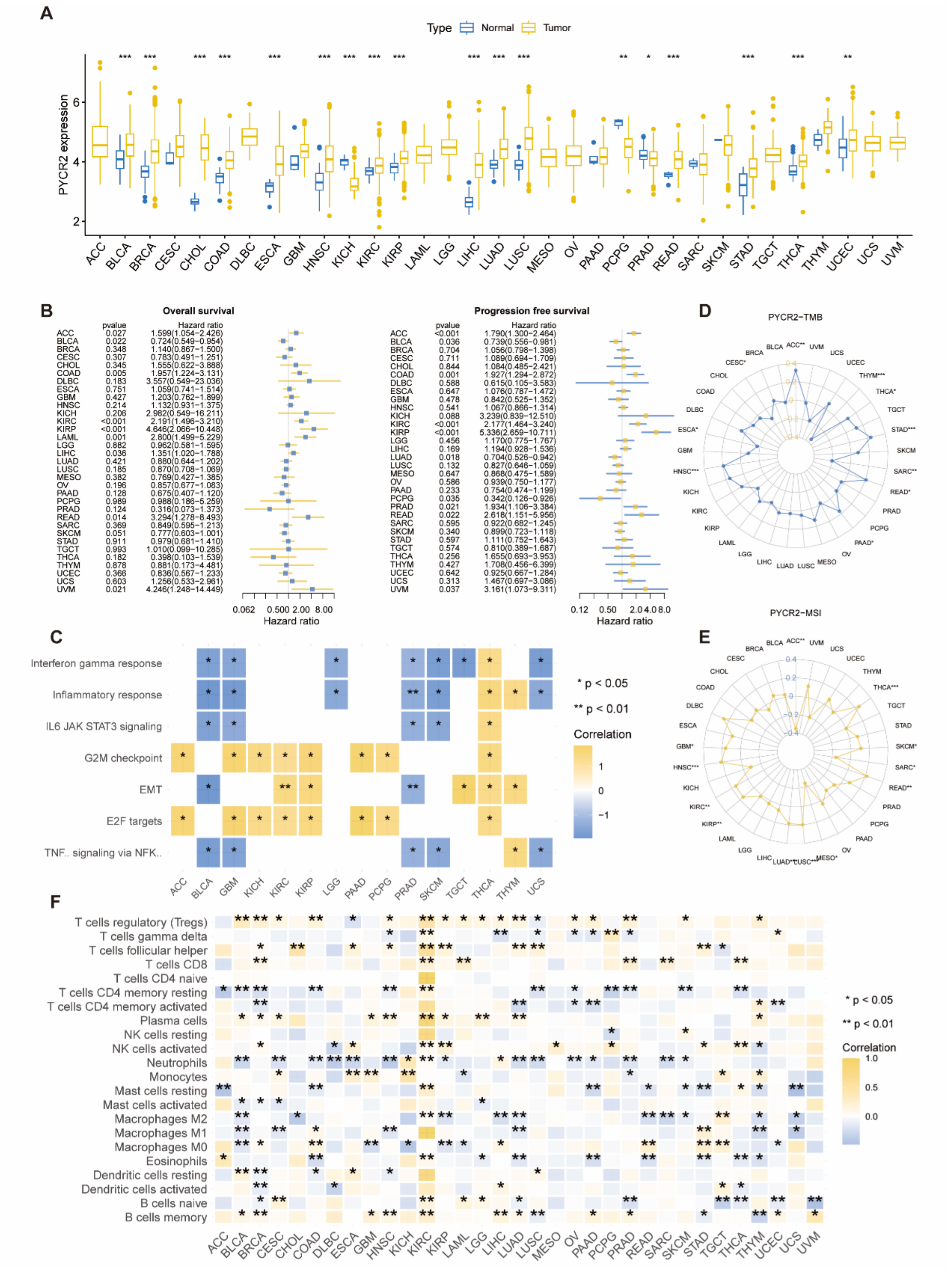

Figure S1: Analysis of PYCR2 in Pan-Cancer. (**A**) Differential expression analysis of PYCR2; (**B**) results of single-factor Cox regression analysis of PYCR2 with prognosis (OS and PFS); (**C**) visualization of seven pathways enriched by PYCR2 in multiple tumors; (**D,E**) analysis of the expression of PYCR2 with TMB and MSI; (**F**) analysis of the expression of PYCR2 with the infiltration of 22 immune cells; * $p < 0.05$, ** $p < 0.01$, and *** $p < 0.001$.

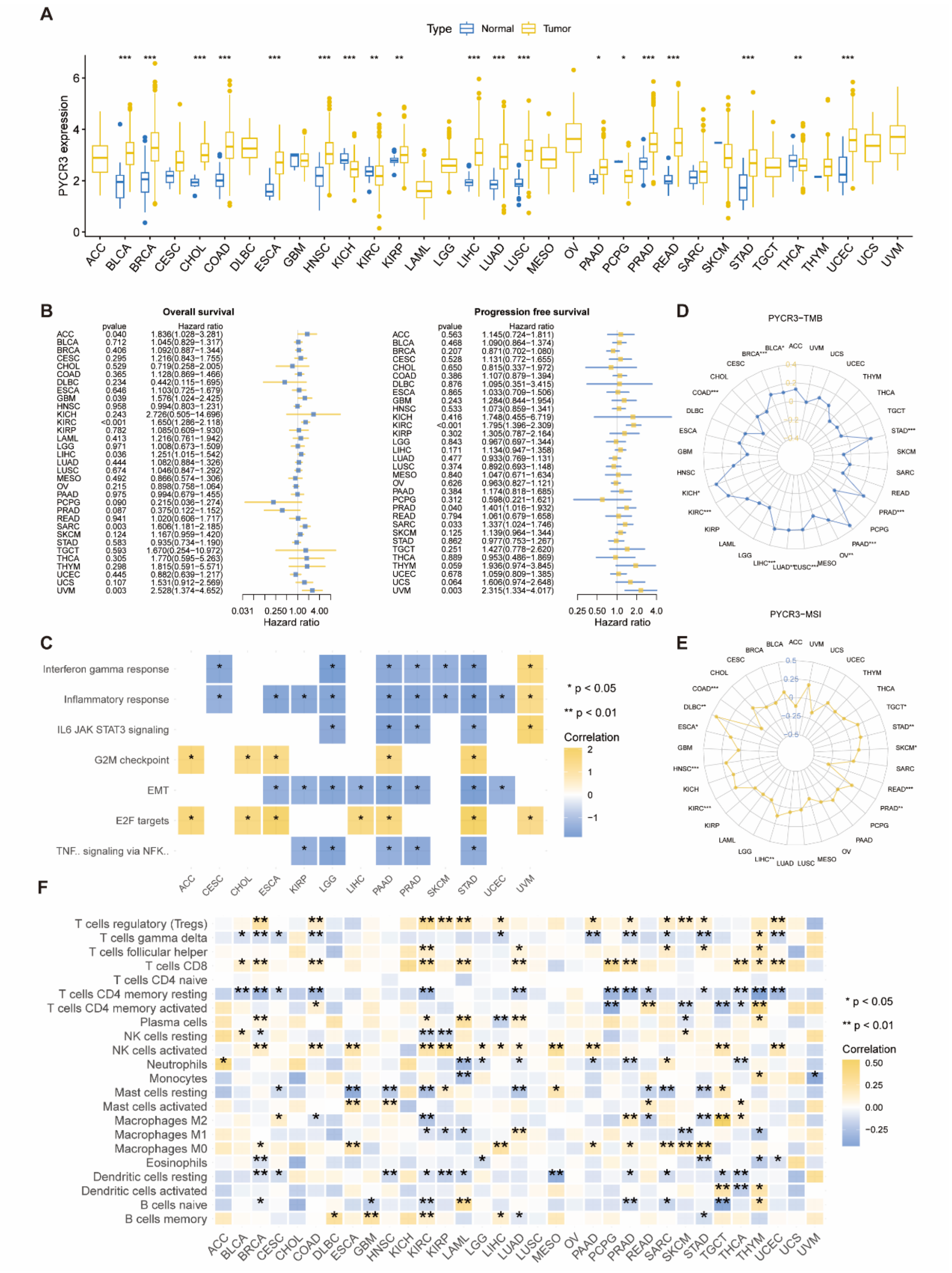

Figure S2: Analysis of PYCR3 in Pan-Cancer. (**A**) Differential expression analysis of PYCR3; (**B**) results of single-factor Cox regression analysis of PYCR3 with prognosis (OS and PFS); (**C**) visualization of seven pathways enriched by PYCR3 in multiple tumors; (**D**,**E**) analysis of the expression of PYCR3 with TMB and MSI; (**F**) analysis of the expression of PYCR3 with the infiltration of 22 immune cells; * $p < 0.05$, ** $p < 0.01$, and *** $p < 0.001$.

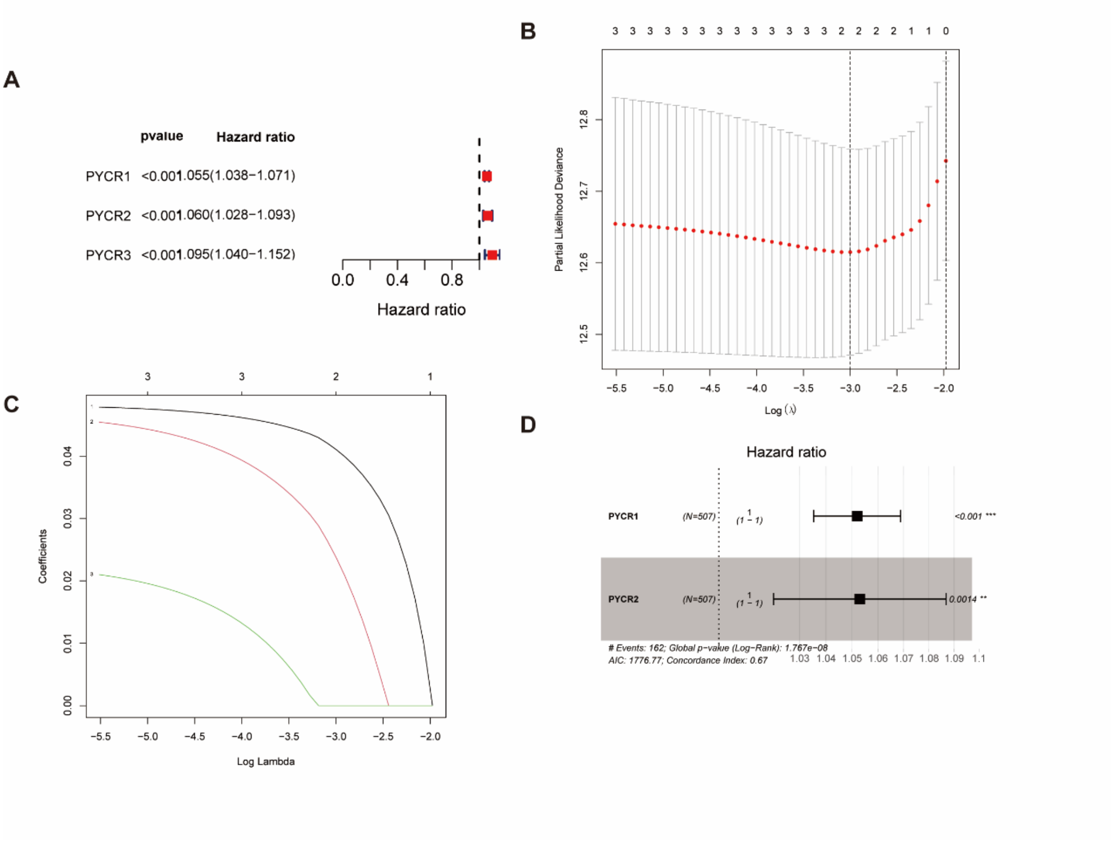

Figure S3: Construction of Prognostic Risk Score Model for KIRC Patients. (**A**): Cox regression analysis of PYCRs for prognosis (OS) in KIRC. (**B**): Confidence intervals for each λ. (**C**): Construction of the LAS-SO regression model. (**D**): Forest plot of the results of the multivariate Cox regression analysis determining the use of PYCR1 and PYCR2 for building the risk score model.

2. Supplementary Tables

Table S1. Clinical characteristics of patients with TCGA-KIRC in each data set

Variables	Total (n = 407)	Train (n = 254)	Tnternal validation (n = 253)	p
Risk, n (%)				
high	253 (50)	128 (50)	125 (49)	0.8939
low	254 (50)	126 (50)	128 (51)	
Age, n (%)				
<60	240 (47)	121 (48)	119 (47)	0.9626
>=60	267 (53)	133 (52)	134 (53)	
Gender, n (%)				
Female	177 (35)	85 (33)	92 (36)	0.5542
Male	330 (65)	169 (67)	161 (64)	
Grade, n (%)				
G1/G2	227 (45)	117 (46)	110 (43)	0.62

G3/G4/GX	280 (55)	137 (54)	143 (57)	
Stage, n (%)				
Stage I/II	306 (60)	148 (58)	158 (62)	0.3833
Stage III/IV	201 (40)	106 (42)	95 (38)	
T_stage, n (%)				
T1/T2	324 (64)	155 (61)	169 (67	0.2072
T3/T4	183 (36)	99 (39)	84 (33)	
N_stage, n (%)				
N0	225 (44)	104 (41)	121 (48)	0.1416
N1/NX	282 (56)	150 (59)	132 (52)	
M_stage, n (%)				
M0	404 (80)	207 (82)	197 (78)	0.3652
M1/MX	103 (20)	47 (18)	56 (22)	

Table S2. Clinical characteristics of patients with TCGA-KIRC in Pathomics Dataset

Variables	Total (n = 407)	Train (n = 204)	Validation (n = 203)	p
PYCR, n (%)				1
low	210 (52)	105 (51)	105 (52)	
high	197 (48)	99 (49)	98 (48)	
Age, n (%)				0.804
<60	203 (50)	100 (49)	103 (51)	
>=60	204 (50)	104 (51)	100 (49)	
Gender, n (%)				0.419
Female	135 (33)	72 (35)	63 (31)	
Male	272 (67)	132 (65)	140 (69)	
Grade, n (%)				0.515
G1/G2	186 (46)	97 (48)	89 (44)	
G3/G4/GX	221 (54)	107 (52)	114 (56)	
Stage, n (%)				0.378
Stage I/II	253 (62)	122 (60)	131 (65)	
Stage III/IV	154 (38)	82 (40)	72 (35)	
T_stage, n (%)				0.153
T1/T2	268 (66)	127 (62)	141 (69)	
T3/T4	139 (34)	77 (38)	62 (31)	
M_stage, n (%)				0.323
M0	330 (81)	161 (79)	169 (83)	

M1/MX	77 (19)	43 (21)	34 (17)	
N_stage, n (%)				0.806
N0	191 (47)	94 (46)	97 (48)	
N1/NX	216 (53)	110 (54)	106 (52)	

Table S3 Target sequences of related gene

Gene Symbol		sense（5'-3'）	antisense（5'-3'）	Nt (bp)
PYCR1	siRNA1	GCCCACAAGAUAAUGGCUATT	UAGCCAUUAUCUUGUGGGCTT	520
	siRNA2	GAAGAAGCUGUCAGCGUUUTT	AAACGCUGACAGCUUCUUCTT	758
	siRNA3	CUAACUUGCUCUCGAUCCUTT	AGGAUCGAGAGCAAGUUAGTT	358
PYCR2	siRNA1	GCCAGCUUAAGGACAAUGUTT	ACAUUGUCCUUAAGCUGGCTT	812
	siRNA2	CCCUCUUAGACAGAGUGAATT	UUCACUCUGUCUAAGAGGGTT	992
	siRNA3	CUGUGGAGAAGAAGCUGAUTT	AUCAGCUUCUUCUCCACAGTT	446

Table S4. Evaluation of the Performance of the pathomics feature model

name	Train_acc	Train_sens	Train_spec	Train_ppv	Train_npv	train_brierScore
PYCR_cat_PS	0.765	0.667	0.857	0.815	0.732	0.178
name	Test_acc	Test_sens	Test_spec	Test_ppv	Test_npv	test_brierScore
PYCR_cat_PS	0.749	0.612	0.876	0.822	0.708	0.208
